# Ambient Air Pollution and Risk of Congenital Anomalies: A Systematic Review and Meta-analysis

**DOI:** 10.1289/ehp.1002946

**Published:** 2010-12-03

**Authors:** Martine Vrijheid, David Martinez, Sandra Manzanares, Payam Dadvand, Anna Schembari, Judith Rankin, Mark Nieuwenhuijsen

**Affiliations:** 1 Center for Research in Environmental Epidemiology, Barcelona, Spain; 2 Hospital del Mar Research Institute (IMIM), Barcelona, Spain; 3 CIBER Epidemiología y Salud Pública, Spain; 4 Educational Unit of Preventive Medicine and Public Health, Hospital del Mar–Pompeu Fabra University–Agencia de Salut Pública, Barcelona, Spain; 5 Institute of Health and Society, Newcastle University, Newcastle upon Tyne, United Kingdom

**Keywords:** air pollution, congenital abnormalities, pregnancy

## Abstract

**Objective:**

We systematically reviewed epidemiologic studies on ambient air pollution and congenital anomalies and conducted meta-analyses for a number of air pollutant–anomaly combinations.

**Data sources and extraction:**

From bibliographic searches we extracted 10 original epidemiologic studies that examined the association between congenital anomaly risk and concentrations of air pollutants. Meta-analyses were conducted if at least four studies published risk estimates for the same pollutant and anomaly group. Summary risk estimates were calculated for *a*) risk at high versus low exposure level in each study and *b*) risk per unit increase in continuous pollutant concentration.

**Data synthesis:**

Each individual study reported statistically significantly increased risks for some combinations of air pollutants and congenital anomalies, among many combinations tested. In meta-analyses, nitrogen dioxide (NO_2_) and sulfur dioxide (SO_2_) exposures were related to increases in risk of coarctation of the aorta [odds ratio (OR) per 10 ppb NO_2_ = 1.17; 95% confidence interval (CI), 1.00–1.36; OR per 1 ppb SO_2_ = 1.07; 95% CI, 1.01–1.13] and tetralogy of Fallot (OR per 10 ppb NO_2_ = 1.20; 95% CI, 1.02–1.42; OR per 1 ppb SO_2_ = 1.03; 95% CI, 1.01–1.05), and PM_10_ (particulate matter ≤ 10 μm) exposure was related to an increased risk of atrial septal defects (OR per 10 μg/m^3^ = 1.14; 95% CI, 1.01–1.28). Meta-analyses found no statistically significant increase in risk of other cardiac anomalies and oral clefts.

**Conclusions:**

We found some evidence for an effect of ambient air pollutants on congenital cardiac anomaly risk. Improvements in the areas of exposure assessment, outcome harmonization, assessment of other congenital anomalies, and mechanistic knowledge are needed to advance this field.

There is growing epidemiologic evidence for adverse effects on the fetus and newborn from maternal prenatal exposure to ambient air pollution (Glinianaia et al. 2004b; Maisonet et al. 2004; Šrám et al. 2005). Air pollutants such as carbon monoxide (CO), sulfur dioxide (SO_2_), and particulate matter (PM) have been associated with increased infant mortality, particularly postneonatal respiratory mortality, low birth weight, and preterm birth (Glinianaia et al. 2004a, 2004b; Maisonet et al. 2004; Šrám et al. 2005). Inconsistencies and uncertainties remain concerning the effects of specific pollutants and pollutant mixtures and critical exposure periods (Ritz and Wilhelm 2008; Woodruff et al. 2009). There are new concerns that air pollution may also play a role in causing congenital anomalies, and such an effect is both biologically plausible and of public health importance (Ritz 2010). Congenital anomalies are a main cause of infant mortality and an important contributor to childhood and adult morbidity. Major congenital anomalies in surviving infants often have serious medical and/or cosmetic consequences that commonly require surgery and lead to reduced survival rates into adulthood (Tennant et al. 2010). Major structural congenital anomalies are diagnosed in 2–4% of births, but in most cases their etiology remains unknown (Weinhold 2009).

Recently, there has been a steep increase in the number of air pollution studies with congenital anomalies as the primary health outcome. The first publication appeared in 2002 (Ritz et al. 2002), the next in 2005 (Gilboa et al. 2005), and more have followed in the last few years (Dadvand et al. 2011a, 2011b; Dolk et al. 2010; Hansen et al. 2009; Hwang and Jaakkola 2008; Kim et al. 2007; Marshall et al. 2010; Rankin et al. 2009; Strickland et al. 2009). Because the existing studies have few *a priori* hypotheses and tested many different pollutant–outcome combinations, a systematic assessment of the consistency of associations across studies is needed. Furthermore, in this rapidly evolving field, timely evaluation of methods and results of existing studies can help to inform and improve the design of future research. Here we therefore provide a systematic review of studies on ambient air pollution and congenital anomalies and develop recommendations for future research.

There have been few meta-analyses of other environmental exposures and risk of congenital anomalies (e.g., Nieuwenhuijsen et al. 2009) because of great variability in exposure assessment, outcome ascertainment, data analysis, and result reporting. Air pollution is one area where exposure estimates appear reasonably comparable. To illustrate the challenges faced by meta-analyses in this field and to offer recommendations for improvement, we conducted meta-analyses for a number of air pollutant–anomaly combinations.

## Materials and Methods

### Search methods

We followed published guidelines for the reporting of this review and meta-analysis (Moher et al. 2009; Stroup et al. 2000). A bibliographic search was carried out in the MEDLINE engine search (National Library of Medicine 2010). The Medical Subject Heading (MeSH) terms “congenital abnormalities,” “pregnancy,” and “air pollution” and non-MeSH terms “birth defect,” “congenital anomalies,” “cardiac anomalies,” “congenital heart disease,” and “oral clefts” were used in the syntax. We also searched references in published articles and reviews on this topic. From this search we selected articles that *a*) were original epidemiologic studies; *b*) were written in the English language; *c*) defined all or subgroups of congenital anomalies, congenital malformations, or birth defects as outcome; and *d*) studied human prenatal exposure to ambient air pollution using measured concentrations of air pollutants. Studies with purely ecologic exposure assessments (e.g.,maternal residence in a polluted vs. unpolluted area) or studies with quantitative traffic density data but without pollutant data (e.g., Cordier et al. 2004) were not included in the review. We identified one article unpublished at the time of the last MEDLINE search but now published (Dadvand 2011b). We searched conference proceedings in the ISI Web of Science (Thomson Reuters 2010) for abstracts of other unpublished studies, using the same search terms as above, but we found no other studies that fall into this category.

### Meta-analysis

We conducted meta-analyses to obtain summary risk estimates for the association between congenital anomaly groups and ambient air pollutant concentrations. Meta-analyses were conducted only if at least four studies had risk estimates [usually odds ratios (ORs)] for the same pollutant and comparable anomaly group. Although no guidelines exist as to the minimum number of studies for meta-analysis, we considered four the minimum number to justifiably run a meta-analysis; this corresponded to having a minimum of 500 cases of congenital anomaly included in each analysis. A list of the specific outcome groups used in the different studies, and the congenial anomaly groups considered comparable for the purposes of these meta-analyses, is available in Supplemental Material, Table 1 (doi:10.1289/ehp.1002946). Meta-analyses calculated two types of summary risk estimates: *a*) for the comparison of congenital anomaly risk at high versus low exposure level in each study and *b*) for congenital anomaly risk per unit increase in continuous pollutant concentration. For each of these, we selected risk estimates from the main, confounder-adjusted models presented in each study, not those of sensitivity analyses. If both single- and multiple-pollutant models were presented, we selected the single-pollutant model. If studies presented results for more than one pregnancy period (Hwang and Jaakkola 2008; Ritz et al. 2002), we selected the period most appropriate for the development of congenital anomalies and most used in the other studies: month 2 or weeks 3–8 of gestation. The Strickland et al. (2009) study used two groups of ventricular septal defects (VSDs): muscular and perimembranous type. We calculated the combined risk estimate for these two groups and then entered the combined estimate in meta-analyses. Two studies (published in three reports) were located in the same study area, covered overlapping time periods, and analyzed some of the same anomaly groups (VSDs, coarctation of the aorta, tetralogy of Fallot) in relation to SO_2_ exposure (Dadvand et al. 2011a, 2011b; Rankin et al. 2009). We entered risk estimates from these publications separately in the SO_2_ meta-analyses, never at the same time. Because the Dadvand et al. (2011b) substudy had risk estimates for other air pollutants and covered a more recent time period, we used this as the main analysis for SO_2_ and considered the inclusion of the Dadvand et al. (2011a) and Rankin et al. (2009) studies to be sensitivity analyses.

In the high- versus low-exposure meta-analysis, we selected from the published reports risk estimates for the fourth compared with the first quartile of exposure, if categorical risk estimates were presented (Dadvand et al. 2011a, 2011b; Gilboa et al. 2005; Marshall et al. 2010; Rankin et al. 2009; Ritz et al. 2002). Some studies presented risk estimates only for continuous exposure (Hansen et al. 2009; Hwang and Jaakkola 2008; Strickland et al. 2009); we converted these to the risk estimate per interquartile increase in exposure, using the interquartile ranges (IQRs) given in the reports, because these were considered to compare most closely with estimates from the categorical studies. Information to calculate midquartile points was not given in these articles, so we could not compare the 12.5th and 87.5th percentiles. One study (Dolk et al. 2010) presented risk estimates for the 90th versus 10th percentile exposure, calculated from a continuous pollutant model, and this estimate was used for the high versus low comparison.

Meta-analyses for continuous exposure summarized risk estimates per unit increase in pollutant concentration, assuming that the natural logarithm of the relative risk of congenital anomaly varied linearly with the ambient air pollutant concentration. From each publication we selected the risk estimate per continuous unit increase in pollutant concentration, if available (Dadvand et al. 2011a; Dolk et al. 2010; Hansen et al. 2009; Hwang and Jaakkola 2008; Ritz et al. 2002; Strickland et al. 2009). Three articles described continuous exposure estimates in the text but did not show the risk estimates (Dadvand et al. 2011b; Gilboa et al. 2005; Marshall et al. 2010); for these we obtained the continuous estimates directly from the authors. To allow comparison of effects among the different studies, units were converted to 10 μg/m^3^ for particulate matter with diameter ≤ 10 μm (PM_10_), 10 ppb for ozone (O_3_) and nitrogen dioxide (NO_2_), 1 ppm for CO, and 1 ppb for SO_2_. Exposure estimations that were expressed in a mass per volume unit (e.g., micrograms per cubic meter) instead of parts per million, billion, or hundred million (pphm) were converted to parts per billion using the general equation at 1 atm and 25°C: 24.45 × concentration (micrograms per cubic meter)/molecular weight. An exception was made for PM_10_, which was always expressed in micrograms per cubic meter.

We obtained summary risk estimates in meta-analyses using fixed- or random-effects models. For each pollutant–outcome analysis we first tested for heterogeneity in the risk estimates using the *Q*-test (Cochran 1954). When the result of the *Q*-test showed evidence for heterogeneity (*p* < 0.1), we used a random effect analysis, following the method of DerSimonian and Laird (1986). Otherwise, a fixed-effect analysis was conducted using the Mantel–Haenszel method (Mantel and Haenszel 1959). Meta-analyses calculated summary risk estimates (ORs) weighted by the inverse variance of each study, taking into account whether a fixed or random model was used. We used the R statistical software package for all analyses (version 2.11.0; R Project for Statistical Computing 2010).

We also produced forest plots to show ORs from each of the individual studies included in the meta-analyses and the estimation of the summary OR (Light and Pillemer 1984). The sizes of the markers of each OR in the plots represent the relative weight each study contributed to the summary estimation. To analyze potential for publication bias, we conducted a weighted Egger test, a linear regression in which the response is the estimated effect and the explanatory variable is a precision term (1/SE) (Egger et al. 1997). A large deviation from zero of the slope term suggests publication bias.

## Results

We identified 10 studies, one divided into two substudies (Dadvand et al. 2011a, 2011b), published between 2002 and 2011 (Table 1). Four studies were conducted in the United States (in California, Texas, Georgia, and New Jersey), three in England (two in the northern region and one in four English regions including the northern region), and one each in Australia, Taiwan, and South Korea. The studies focused mainly on cardiac anomalies (Dadvand et al. 2011a, 2011b; Gilboa et al. 2005; Hansen et al. 2009; Ritz et al. 2002; Strickland et al. 2009) and/or orofacial clefts (Gilboa et al. 2005; Hansen et al. 2009; Hwang and Jaakkola 2008; Marshall et al. 2010; Ritz et al. 2002). Only two studies (Dolk et al. 2010; Rankin et al. 2009) included the full spectrum of major structural anomalies. The South Korean study was a prospective birth cohort study that included all congenital anomalies as one group (14 cases) (Kim et al. 2007). Most other studies used a registry-based case–control design, selecting cases from routine congenital anomaly registries and controls from birth registries. One study was a registry-based cohort, using anomaly registries as source of case ascertainment and birth registry data for denominators (Dolk et al. 2010). Strickland et al. (2009) used a time-series design to link daily exposure estimates to daily congenital anomaly rates (for a given conception date); again, data on the congenital anomalies came from a routine register.

Major differences are apparent in the diagnostic coding systems, congenital anomaly grouping methods, and case definitions among the studies [see Supplemental Material, Table 1 (doi:10.1289/ehp.1002946)]. The studies by Gilboa et al. (2005) and Hansen et al. (2009) based their groupings of cardiac anomalies and orofacial clefts on the anatomic classification used in the first study by Ritz et al. (2002); inclusion and exclusion of chromosomal, syndromic, and multiple anomalies still differed among these studies. The Georgia study (Strickland et al. 2009) used a more detailed system of diagnostic codes for cardiac anomalies. The U.K. studies (Dadvand et al. 2011a, 2011b; Dolk et al. 2010; Rankin et al. 2009) all used coding and grouping system based on the *International Classification of Diseases, 9th Revision* (World Health Organization 1975) and the minor anomaly exclusion criteria proposed by the European Surveillance of Congenital Anomalies (EUROCAT 2009).

In all but one study (Dadvand et al. 2011a), exposure assessments were based on the routine measurements of air pollutant concentrations at fixed-site air pollution monitoring stations. Most commonly, exposures were assigned using the monitoring station nearest to the maternal residence at the time of the birth. Distances of residence from the monitors varied and in some studies inclusion of study subjects was limited by their distance to the monitor [e.g., 16 km in the Ritz et al. (2002) study, 10 km in the Rankin et al. (2009) study]. Number and density of monitors also varied, as did the maximum distance of subjects from the nearest monitor (Table 1). Dadvand et al. (2011a) developed a spatiotemporal model in which black smoke and SO_2_ concentrations at the maternal residence were predicted using concentrations measured at 56 monitors and data on traffic, meteorology, and land cover. The Georgia study (Strickland et al. 2009) differed from others in that temporal data from one central monitoring station in the county were related to the vulnerable window of each pregnancy in a time-series analysis. Nearly all other studies defined windows of pregnancy susceptible to the development of the congenital anomalies under study (usually weeks 3–8 of gestation) and averaged exposure over those windows. The Dolk et al. (2010) study assigned mean pollutant concentrations measured in one year (1996) to the census ward level of residence and was not able to estimate exposure in pregnancy time windows.

Most studies focused on the most commonly monitored air pollutants: PM_10_ (nine studies), SO_2_ (eight studies), NO_2_ (seven studies), CO (seven studies), and O_3_ (seven studies). Less frequently studied pollutants were black smoke, nitric oxide (NO), nitrogen oxides (NO_x_), and particulate matter with aerodynamic diameter ≤ 2.5 μm (PM_2.5_). Pollutant concentration distributions showed different patterns across studies (Table 1); for example, average CO concentrations were highest in the California study (Ritz et al. 2002), but O_3_ concentrations were highest in the Taiwan study (Hwang and Jaakkola 2008). In most studies where this information was provided, pollutant concentrations did not increase by more than a factor of 2 between the 25th and 75th percentile (Table 1).

### Cardiac anomalies

Cardiac anomalies were analyzed in seven studies. Each study included at least six separate cardiac anomaly groups [see Supplemental Material, Table 1 (doi:10.1289/ehp.1002946)] and tested these against three to six pollutant groups. Each study reported only one or a few statistically significantly increased risks with increased exposure among the multiple associations tested; few of these occurred in more than one study: CO was related to higher risk of VSD in two studies, with ORs for fourth- versus first-quartile exposure of 2.95 [95% confidence interval (CI), 1.44–6.05 (Ritz et al. 2002)] and 1.66 [95% CI, 1.37–2.02 (Dadvand et al. 2011b)]. The studies in California and Australia both reported raised risks of pulmonary artery and valve defects in association with O_3_ exposure [OR quartile 4 vs. quartile 1 = 2.94; 95% CI, 1.00–6.05 (Ritz et al. 2002); OR per 5 ppb = 2.96; 95% CI, 1.34–7.52 (Hansen et al. 2009)]. Various inverse associations (decreasing risks with increasing exposure) were also observed.

**Figure 1:**
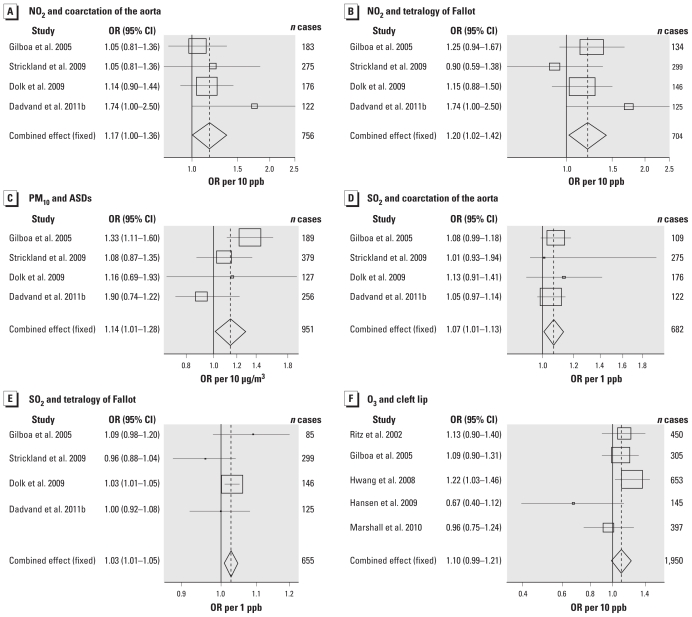
Forest plots showing risk estimates for individual studies and the combined meta-analysis result. Marker sizes represent the relative weight the study contributed to the summary estimation. (*A*) NO_2_ and coarctation of the aorta. (*B*) NO_2_ and tetralogy of Fallot. (*C*) PM_10_ and ASDs. (*D*) SO_2_ and coarctation of the aorta. (*E*) SO_2_ and tetralogy of Fallot. (*F*) O_3_ and cleft lip with or without cleft palate.

We conducted meta-analyses for 18 combinations of pollutants and cardiac anomaly groups for which four or more studies published results [for summary results, see Table 2; for full results, see Supplemental Material, Table 2 (doi:10.1289/ehp.1002946)]. The summary risk estimates from these meta-analyses were generally close to one, with a range of summary ORs for continuous exposure from 0.87 to 1.20, and for high versus low exposure from 0.80 to 1.23. Heterogeneity tests showed evidence for heterogeneity among studies (*p* < 0.10) in fewer than half of the analyses conducted, most consistently related to analyses of VSDs. Egger test *p*-values were statistically significant for only 3 of the 68 meta-analyses we conducted (see Supplemental Material, Table 2), indicating that wide-scale publication bias is unlikely. We found statistically significantly increased summary risk estimates for continuous NO_2_ exposure and risk of coarctation of the aorta (OR per 10 ppb = 1.17; 95% CI, 1.01–1.36) and tetralogy of Fallot (OR per 10 ppb = 1.20; 95% CI, 1.02–1.42), for continuous PM_10_ exposure and atrial septal defect (ASD; OR per 10 μg/m^3^ = 1.14; 95% CI, 1.01–1.28), and for continuous SO_2_ exposure and risk of coarctation of the aorta (OR per 1 ppb = 1.07; 95% CI, 1.01–1.13) and tetralogy of Fallot (OR per 1 ppb = 1.03; 95% CI, 1.01–1.05) (Table 2, Figure 1A–E). *p*-Values for heterogeneity in these analyses showed limited evidence for heterogeneity (*p*-values between 0.1 and 0.9). Sensitivity analyses excluding the study with the largest weight from each meta-analysis showed that results for NO_2_ and tetralogy of Fallot and for SO_2_ and coarctation of the aorta were robust to this exclusion. The results for SO_2_ were not robust to the inclusion of the first Dadvand study (Dadvand et al. 2011a) instead of the second (Dadvand et al. 2011b) (Table 2); the first study by Dadvand et al. (2011a) also introduced significant heterogeneity. High versus low comparisons did not show evidence for increased risks for any anomaly–pollutant combinations. The summary OR for high compared with low CO exposure and risk of ASDs was significantly reduced (OR per 1 ppm = 0.86; 95% CI, 0.75–0.99).

### Orofacial clefts

Seven studies examined the association between air pollutants and risk of cleft lip with or without cleft palate (Table 1). Statistically significantly increased risks of cleft lip with or without cleft palate were observed in relation to SO_2_ in two studies [OR per 0.6 ppb = 1.27; 95% CI, 1.01–1.62 (Hansen et al. 2009); OR quartile 4 vs. quartile 1 = 1.6; 95% CI, 1.1–2.2 (Marshall et al. 2010)]. None of the studies reported increased risks of cleft palate alone. Meta-analyses summarizing risk estimates for exposure to five pollutants and risk of cleft lip with or without cleft palate and cleft palate alone were all close to one, and none reached statistical significance (Table 3). We found evidence for significant heterogeneity (*p* < 0.1) in the CO and SO_2_ analyses. O_3_ exposure and cleft lip with or without cleft palate showed an association of borderline statistical significance (OR per 10 ppb = 1.10; 95% CI, 0.99–1.21) (Table 3, Figure 1F).

### Other congenital anomalies

Two studies examined a range of anomalies other than cardiac anomalies and orofacial clefts (Dolk et al. 2010; Rankin et al. 2009). They observed statistically significantly increased risks of omphalocele in relation to PM_10_ concentrations [90th vs. 10th percentile: OR = 2.17; 95% CI, 1.00–4.71 (Dolk et al. 2010)] and of nervous system anomalies in relation to black smoke concentration [OR = 1.10/mg/m^3^; 95% CI, 1.03–1.18 (Rankin et al. 2009)]. No other anomaly groups/subtypes were at an increased risk in relation to the pollutants studied.

## Discussion

The evidence base for an effect of exposure to ambient air pollutants on congenital anomaly risk is small. Individual studies reported increased risks for some combinations of air pollutants and congenital anomalies, mostly cardiac anomalies, but these occurred among many associations tested in each study. Meta-analyses suggest that NO_2_ and SO_2_ exposures were related to statistically significant increases in risk of coarctation of the aorta and tetralogy of Fallot, and PM_10_ exposure to an increase in risk of ASDs; we based summary risk estimates on few studies (*n* = 4), but the total numbers of cases included were relatively large (between 655 and 951). Meta-analyses found no statistically significant increase in risk of other cardiac anomalies and orofacial clefts in relation to air pollution exposure. This review and its meta-analysis raise important issues that may guide both the design and presentation of future studies.

A common feature of all the reviewed studies was the use of routine monitoring stations as the basis for exposure assessment. Exposure indices were usually calculated from pollutant measurements at the nearest monitoring station or as a distance-weighted average of measurements of all stations in the area; these methods apply a similar exposure to a relatively large geographic area and thereby measure predominantly community-wide variations in air pollution. This approach will be more appropriate for pollutants that vary at large geographic scale (e.g., SO_2_, O_3_) than for those that may have a much finer spatial distribution/resolution (e.g., CO, NO_2_). Traffic exhaust fumes are the main source of air pollutants such as NO_2_ and PM_2.5_ in urban areas, as well as a main source of suspected causative agents for adverse birth outcomes, such as polycyclic aromatic hydrocarbons (PAHs) (Perera et al. 2003; Ritz and Wilhelm 2008). More precise spatial models, based on dispersion or land-use regression models that take into account local road networks and other predictive variables, are therefore increasingly being recommended and used in research on adverse birth outcomes (Aguilera et al. 2009; Gilliland et al. 2005; Madsen et al. 2010; Nethery et al. 2008; Ritz and Wilhelm 2008; Slama et al. 2007, 2008; Wu et al. 2009). Only one of the reviewed studies (Dadvand et al. 2011a) used a spatiotemporal model for black smoke and SO_2_ exposure in which concentrations of these pollutants were predicted from monitoring data combined with data from traffic, meteorology, and land use. Application of these types of models to future studies of congenital anomalies would be a step toward more accurate exposure assessment for some of the pollutants of interest. Characterization and quantification of errors in exposure estimates from these models will be available from validation studies (Nethery et al. 2008), and efforts should be made to also incorporate these in evaluations of congenital anomaly risk. Furthermore, air pollution studies of other birth outcomes have moved toward trying to estimate more specifically the exact pollutants responsible (e.g., transition metals contained in PM, PAHs, aromatic hydrocarbons) (e.g., Aguilera et al. 2009; Perera et al. 2003; Slama et al. 2007), rather than focusing on the regulated pollutants, and this would be a valuable direction for future congenital anomaly studies.

Nearly all the reviewed studies were based on registry information. In such studies, residential addresses are available only at birth, not in the first trimester of pregnancy, the most relevant period for causation of congenital anomalies (Moore and Persaud 1998). Furthermore, residential addresses only account for exposures near the home, not in other situations thought to make an important contribution to personal exposure, such as work location, commuting, and indoor air pollution sources (Nethery et al. 2008; Setton et al. 2010). However, congenital anomaly research in this field cannot easily move away from routinely registered data. Large case–control studies with good information on residential history and time–activity patterns may be a useful step forward, if they can be combined with accurate exposure assessments. Pregnancy cohort studies in Europe are currently pooling resources to study effects of air pollution and other birth outcomes using land-use regression exposure models [European Study of Cohorts for Air Pollution Effects (ESCAPE) Project 2009], but few such cohorts are large enough to conduct meaningful analyses of congenital anomalies or include thorough enough ascertainment of congenital anomalies among pregnancy terminations as well as live births. Information from such studies may, however, provide useful information about the effect of including information on residential mobility and time–activity patterns on risk estimates for other birth outcomes (e.g., Aguilera et al. 2009; Chen et al. 2010; Lupo et al. 2010).

The most difficult issue in our attempt to combine data from the different studies was the use of very different criteria for definition and classification of congenital anomaly subgroups. Moreover, we found differences in inclusion and exclusion of cases with other anomalies in the same and other organ systems, with chromosomal abnormalities, and with other syndromic conditions. We compared the different groupings and exclusions used [see Supplemental Material, Table 1 (doi:10.1289/ehp.1002946)] but found that in some instances it is not possible to deduce from the articles which inclusions and exclusions were made. For our meta-analyses we selected a few groups of relatively comparable anomaly groups. However, even for these, studies differed in their approach to classification. VSDs, for example, were treated as one anomaly group by some studies (Dadvand et al. 2011a, 2011b; Gilboa et al. 2005; Hansen et al. 2009; Rankin et al. 2009; Ritz et al. 2002), as four different anomalies by another (Strickland et al. 2009), and were excluded in yet another (Dolk et al. 2010). Notably, we found the largest evidence for heterogeneity for VSDs. In general, congenital heart defects form a very heterogeneous set of conditions, notoriously difficult to classify (Botto et al. 2007; Strickland et al. 2008). Several classification systems have been proposed (Botto et al. 2007; Jacobs et al. 2007), but diagnostics information in routine registries may often not be specific enough to apply these. Further international harmonization efforts in this area [e.g., those undertaken as part of EUROCAT (2010) and the International Clearinghouse for Birth Defects (2010)] will be of great value. Classification of orofacial clefts is more straightforward than that of cardiac anomalies, but even here differences in exclusions have resulted in somewhat nonhomogeneous case groups for the meta-analyses. We encourage future studies to base their classifications and exclusions on those of previous studies in the same field, where possible.

The meta-analysis results should be interpreted with caution because they are based on few studies, and because some were subject to some degree of statistical heterogeneity. On the other hand, the total numbers of cases included in the meta-analyses were large, ranging from 500 to > 3,700, depending on the anomaly group. We found significant results for some pollutant–cardiac anomaly combinations, but only two of these (NO_2_ and tetralogy of Fallot, and SO_2_ and coarctation of the aorta) were robust to the exclusion of the study with the largest weight, and we could not confirm significant findings from the continuous exposure analyses with high- versus low-exposure analyses. It is not clear which of these latter analyses is the most appropriate: In continuous analyses one assumes a (log) linear relationship between exposure and outcome; in the field of congenital anomaly, linearity cannot automatically be assumed, because selective survival of more viable fetuses related to the same exposure may lead to nonlinearity (Ritz 2010). On the other hand, the high versus low analysis combines categories of very different exposure levels across studies and has lower power because of smaller numbers of cases in these more extreme exposure categories. We recommend that studies report both types of analyses, in annexes of sensitivity analyses where appropriate, in order to aid future meta-analyses.

Heterogeneity in the studies we reviewed may arise from inherent differences between the study settings, as well as from differences in study designs and analysis methods. First, the study areas were different with respect to exposure levels and ranges, pollutant mixtures, and underlying anomaly risks; this may have given rise to different dose–response relationships. With respect to study design, we considered the assessment of exposure to be relatively similar: Studies assessed mostly the same pollutants, same exposure windows, and similar distance-based exposure indices. Heterogeneity in our analyses was not particularly related to studies that deviated from the rest with respect to their approach to exposure assessment such as the Strickland et al. (2009) study with a purely temporal exposure model and the Dolk et al. (2010) study with a purely spatial model. As described above, ascertainment and classification of congenital anomalies differed greatly among studies, and this may well have played a role in the observed heterogeneity. Because all reviewed studies used routinely registered data, selection and recall biases likely did not play a large role in most of the reviewed studies. However, two of the reviewed studies (Gilboa et al. 2005; Hansen et al. 2009) matched controls to cases by, respectively, mother’s county of residence and mother’s distance of residence from an air pollution monitor. This may introduce bias by reducing exposure contrast between cases and controls.

Covariates included in analyses differed and residual confounding structures may differ among the studies, thus leading to heterogeneity in study results. However, the number of known risk factors for congenital anomalies is extremely small, and none of the suspected risk factors, such as smoking, alcohol, folate deficiency, or socioeconomic status, are likely to explain a large proportion of cases. Maternal smoking, for example, has been established as a risk factor for orofacial clefts with relative risk estimates around 1.2–1.3 (Lie et al. 2008; Little et al. 2004; Meyer et al. 2004), but evidence for an association with risk of other congenital anomalies is weak (EUROCAT 2004). Only two of the reviewed studies controlled for tobacco smoking (Gilboa et al. 2005; Marshall et al. 2010).

Possible mechanisms of teratogenicity of air pollutants at this stage remain speculative, but several mechanisms have been hypothesized for effects of PM on fetal growth, including oxidative stress, placental inflammation, and changes in coagulation, as reviewed by Kannan et al. (2006). Congenital anomalies may be induced through similar effects on early fetal growth, as well as by air pollutants influencing the migration and differentiation of neural crest cells, by interactions with the metabolism and detoxification of other xenobiotics, or by indirect effects through maternal immunologic reactions, such as infection or asthma, or medication related to these conditions (Dolk et al. 2010; Ritz et al. 2002; Sram et al. 2005). In animal studies, maternal exposure to O_3_, NO_2_, and CO has produced embryotoxic effects, as well as teratogenic effects such as skeletal and neuromuscular anomalies (Garvey and Longo 1978; Kavlock et al. 1979; Longo 1977; Singh 1988).

Genetic polymorphisms in developmental and detoxification genes have been described as potentially increasing individual susceptibility to the teratogenic effects of maternal smoking (e.g., Chevrier et al. 2008; Lammer et al. 2005); evaluation of similar gene–environment interactions may aid mechanistic understanding in future studies of air pollution and congenital anomalies. Interactions between PM and specific micro- and macronutrients in the diet (antioxidants, antiinflammatory factors) have been proposed to play a role in the causation of low birth weight, fetal growth restriction, and preterm birth, through joint oxidative stress and inflammatory mechanisms (Jedrychowski et al. 2010; Kannan et al. 2006); such interactions also warrant exploration in the causation of congenital anomalies.

## Recommendations

This review of 10 studies finds some evidence of an effect of air pollutants on congenital anomaly risk, so it is worth considering directions for further research in this area. Air pollution is a ubiquitous exposure, and small increases in risk may therefore carry a large public health implication; congenital anomalies are rare but serious outcomes that have a large impact on infant mortality and morbidity and on morbidity in later life. The statistically significant increases in relative risk we observed in meta-analyses were between 3% and 20% per unit of exposure. Exposure units used were of the same order of magnitude as, or well within, the IQRs of these pollutants in most study areas (Table 1). Therefore, relative risk increases of this order of magnitude may be considered important for public health. Existing studies have so far not used some of the recent advances in exposure assessment used in other areas of air pollution research. A case can thus be made for well-designed studies that can make improvements in at least some of the following areas.

### Exposure assessment

As outlined above, exposure indices with better spatial accuracy (while maintaining an accurate temporal component) should be used to assess exposure to traffic-related air pollution, because this is the main source of air pollution in urban areas. Better identification of the effects of specific exposures and exposure mixtures, and the integration of mobility and time–activity patterns, is recommended for congenital anomaly research in the same way as for other birth outcomes. Matching of cases and controls by exposure-related variables such as maternal area of residence should be avoided in future studies.

### Outcome harmonization

International harmonization of coding and classification of anomalies would greatly aid future systematic evaluations in this field. Any future studies should carefully report the exact classifications and exclusions used and attempt to use the same classifications as previous studies, at least in sensitivity analyses.

### Other congenital anomalies

Many of the above studies focused on cardiac anomalies and orofacial clefts. These are two of the largest anomaly groups, and they have been suspected to be related to other environmental exposures (Dolk and Vrijheid 2003). However, studies with sufficient statistical power should also focus on other anomalies for which there may be an environmental etiology (e.g., neural tube defects, limb defects, gastroschisis) (EUROCAT 2004).

### Mechanisms and individual susceptibility

Improved knowledge on potential mechanisms of teratogenic action of air pollutants is needed; this may be achieved by the integration of mechanistic biomarkers. Evaluation of interactions with genetic polymorphisms and dietary factors may also provide further insights into mechanisms.

## Figures and Tables

**Table 1:** Studies on air pollution and risk of congenital anomalies.

Reference	Setting	Design	Case and control definition	Exposure assessment	Exposure range in second month of gestation	Gestational period	Adjustment variables
Ritz et al. 2002	California (USA), four counties, 1987–1993; 754,030 births	Case–control	3,121 cases with cardiac or oral facial cleft defects; live births and fetal deaths > 20 weeks gestations; 9,357 controls, randomly selected from all births and fetal deaths	Average of 24-hr measurements of CO, NO_2_, O_3_, and PM_10_ at nearest monitoring station (within 16 km)	p25–p75: CO, 1.14–2.39 ppm O_3_, 1.07–2.86 pphm	Months 1, 2, and 3; trimesters 2 and 3; 3 months preconception	Maternal age, race, education, prenatal care, infant sex, decade of birth, parity, birth type, season of conception, and other air pollutants
Gilboa et al. 2005	Texas (USA), seven counties, 1997–2000; 607,500 births	Case–control	1,719 cases with cardiac defects or oral clefts; live births and fetal deaths > 20 weeks gestation; 3,667 controls, live births, and fetal deaths, frequency matched by vital status, year of birth, and county	Average hourly or daily concentrations of CO, NO_2_, O_3_, PM_10_, and SO_2_, measured at the nearest, or next nearest, monitor in the county (median distance to monitor, 8.6–14.2 km; maximum, 36–54 km)	p25–p75: CO, 0.4–0.7 ppm NO_2_, 1.3–2.1 pphm O_3_, 1.8–3.1 pphm PM_10_, 19.5–29 μg/m^3^ SO_2_, 1.3–2.7 ppb	Weeks 3–8	Maternal age, education, race/ethnicity, marital status, illness, tobacco use, season of conception, plurality, parity, infant sex, prenatal care, and gravidity
Kim et al. 2007	Seoul (South Korea), 2001–2004; 1,514 births	Birth cohort	14 cases with structural defects in cohort of 1,514 births	Average PM_10_ concentration from nearest of 27 monitoring stations	Mean: PM_10_, 89.7 μg/m^3^	Trimesters 1, 2, and 3	Infant sex, birth order, season of birth, maternal age, education, alcohol, and body mass index
Hwang and Jaakkola 2008	Taiwan, 2001–2003; 721,289 births	Case–control	653 cases with cleft lip with or without cleft palate diagnosed at birth; 6,530 controls randomly selected from all live births	Monthly average of continuous concentration of CO, NO_x_, O_3_, PM_10_, and SO_2_, measured at 72 monitoring stations in Taiwan; inverse distance-weighted average assigned to each residence	p25–p75: CO, 0.48–0.76 ppm NO_x_, 16.0–23.9 ppb O_3_, 24.4–30.1 pphm PM_10_, 44.8–64.5 μg/m^3^ SO_2_, 2.4–5.0 ppb	Months 1, 2, and 3	Maternal age, plurality, gestational age, population density, and season of conception
Rankin et al. 2009	Northern region (UK), 1985–1990; 242,268 births	Case–control	2,779 cases with congenital anomaly; live births and fetal deaths ≥ 20 weeks gestation; 15,000 controls randomly selected from all live and stillbirths	Average of daily black smoke and SO_2_ from all monitoring stations within 10 km of maternal residence	p25–p75: SO_2_, 2.7–4.5 μg/m^3^ Black smoke, 1.1–2.8 μg/m^3^	First trimester	Birth weight, sex, and material deprivation
Strickland et al. 2009	Georgia (USA), five counties, 1986–2003; 715,500 births	Time series	3,338 cases with an indication of cardiovascular malformation; all live births, stillbirths, and fetal deaths > 20 weeks	Average of daily measurements of CO, NO_2_, O_3_, PM_10_, and SO_2_ from one central monitoring station; daily time-series analysis using conception dates	IQR: CO, 0.3 ppm NO_2_, 5.7 ppb O_3_, 3.0 pphm PM_10_, 14.2 μg/m^3^ SO_2_, 4.0 ppb	Weeks 3–7	Week of year, and cubic spline for day of follow-up
Hansen et al. 2009	Brisbane (Australia), 1997–2004; 150,308 births	Case–control	Cases with cardiac defects and clefts, stillbirths and live births; five matched controls per case	Average of daily measurements of CO, NO_2_, O_3_, PM_10_, and SO_2_ at the monitoring site nearest to the center of small area of residence	IQR, mean: CO, 0.6, 1.1 ppm NO_2_, 4.0, 8.2 ppb O_3_, 0.5, 2.6 pphm PM_10_, 4.0, 18.0 μg/m^3^ SO_2_, 0.6, 1.5 ppb	Weeks 3–8	Infant sex; matching variables: maternal age, marital status, indigenous status, parity, month of last menstrual period, area-level socioeconomic status, and distance to monitor
Dolk et al. 2010	Four regions of England, 1991–1999; 759,993 births	Cohort	9,085 cases with chromosomal and nonchromosomal anomalies, live births and fetal deaths ≥ 20 weeks; all live and stillbirths	Annual mean concentrations in 1996 of NO_2_, PM_10_, and SO_2_ for 1 × 1 km grids; population-weighted average assigned to census ward of residence	p10–p90: NO_2_, 21.5–47.8 μg/m^3^ PM_10_, 18.8–26.4 μg/m^3^ SO_2_, 3.9–15.0 μg/m^3^	1996 average	Maternal age, deprivation index, region, and hospital catchment
Marshall et al. 2010	New Jersey (USA), 1998–2003; 690,000 births	Case–control	717 cases with cleft lip and/or palate, live births; random sample of eligible nonmalformed births	Average of measurements of CO, NO_2_, O_3_, PM_10_, PM_2.5_, and SO_2_ at nearest monitor (maximum, 40 km; median, 13–20 km)	p25–p75: CO, 0.65–1.02 ppm NO_2_, 18–30 ppb O_3_, 1.5–3.3 pphm PM_10_, 22–33.5 μg/m^3^ SO_2_, 3–7 ppb	Weeks 3–8	Maternal race, age, education, gravidity, alcohol use, smoking, season of conception, and infant sex
Dadvand et al. 2011a	Northern region (UK), 1985–1996; 449,355 births	Case–control	2,713 cases with cardiac defects, live births and fetal deaths ≥ 20 weeks gestation; 9,975 controls, live and stillbirths	Two-stage spatiotemporal modeling of weekly averages of black smoke and SO_2_ levels (from 56 monitors) at maternal residence using traffic data, land cover data, and other predictors	p25–p75: Black smoke, 5.93–14.57 μg/m^3^ SO_2_, 17.6–31.2 μg/m^3^	Weeks 3–8	Year of birth, socioeconomic status, infant sex, season of conception, and degree of urbanity
Dadvand et al. 2011b	Northern region (UK), 1993–2003; 356,767 births	Case–control	2,140 cases with cardiac defects, live births and fetal deaths > 20 weeks gestation; 14,256 controls, live and stillbirths	Weekly average measurements of CO, NO, NO_2_, O3, PM_10_, and SO_2_, at nearest of six monitoring stations in the region (maximum, 56–83 km; median, 8–12 km)	p25–p75: CO, 0.39–0.64 mg/m^3^ NO_2_, 29.2–38.4 μg/m^3^ NO, 13.3–32.5 μg/m^3^ O_3_, 33.2–42.4 μg/m^3^ PM_10_, 20.5–30.2 μg/m^3^ SO_2_, 6.8–15.0 μg/m^3^	Weeks 3–8	Year of birth, socioeconomic status, infant sex, season of conception, and degree of urbanity

Abbreviations: p10, 10th percentile; p25, 25th percentile; p75, 75th percentile; p90, 90th percentile.

**Table 2:** Summary of meta-analysis of studies on air pollutant exposures and cardiac anomalies.

Pollutant and anomaly combination	Studies included^a^	Total number of cases^b^	Continuous exposure^c^		High versus low exposure	
			Heterogeneity *p*-value	Summary OR (95% CI)^d^	Heterogeneity *p*-value	Summary OR (95% CI)^d^
CO				per 1 ppm		

ASDs	1, 2, 5, 6, 9	1,337	0.10	0.87 (0.72–1.05)	0.17	0.86 (0.75–0.99)
VSDs	1, 2, 5, 6, 9	3,710	< 0.001	1.14 (0.70–1.85)	< 0.001	1.18 (0.82–1.69)
Conotruncal defects	1, 2, 5, 6	1,156	0.02	0.95 (0.57–1.58)	0.01	0.95 (0.62–1.44)

NO_2_				per 10 ppb		

ASDs	2, 5, 6, 9	952	0.81	1.10 (0.91–1.33)	0.28	1.07 (0.90–1.26)
VSDs	2, 5, 6, 9	3,460	0.002	1.12 (0.87–1.44)	0.03	0.92 (0.77–1.12)
Coarctation of the aorta	2, 5, 7, 9	756	0.31	1.17 (1.00–1.36)	0.13	1.04 (0.86–1.26)
Tetralogy of Fallot	2, 5, 7, 9	704	0.22	1.20 (1.02–1.42)	0.06	1.04 (0.70–1.55)

O_3_				per 10 ppb		

ASDs	1, 2, 5, 6, 9	1,307	0.08	1.10 (0.92–1.32)	0.31	0.99 (0.83–1.19)
VSDs	1, 2, 5, 6, 9	3,557	0.02	0.95 (0.83–1.08)	0.02	0.93 (0.73–1.18)
Conotruncal defects	1, 2, 5, 6	1,164	0.64	1.07 (0.96–1.19)	0.45	1.13 (0.89–1.42)

PM_10_				per 10 μg/m^3^		

ASDs	2, 5, 6, 9	951	0.10	1.14 (1.01–1.28)	0.02	1.23 (0.91–1.67)
VSDs	2, 5, 6, 9	3,410	0.12	1.03 (0.96–1.10)	0.70	0.93 (0.84–1.02)
Coarctation of the aorta	2, 5, 7, 9	761	0.02	1.10 (0.88–1.39)	0.48	1.00 (0.79–1.26)
Tetralogy of Fallot	2, 5, 7, 9	546	0.37	1.00 (0.87–1.15)	0.02	0.91 (0.53–1.56)

SO_2_				per 1 ppb		

ASDs	2, 5, 6, 9	909	0.01	0.96 (0.86–1.07)	0.005	1.21 (0.82–1.79)
	2, 5, 6, 10^e^	914	0.01	0.97 (0.88–1.07)	0.02	1.27 (0.91–1.77)
VSDs	2, 5, 6, 9	3,217	0.002	1.04 (0.95–1.15)	< 0.001	0.96 (0.63–1.46)
	2, 5, 6, 10^e^	3,056	< 0.001	1.02 (0.91–1.14)	< 0.001	1.08 (0.81–1.44)
	2, 4, 5, 6^f^				< 0.001	1.05 (0.76–1.46)
Coarctation of the aorta	2, 5, 7, 9	682	0.90	1.07 (1.01–1.13)	0.29	1.06 (0.89–1.27)
	2, 5, 7, 10^e^	687	0.02	1.02 (0.91–1.15)	0.01	0.89 (0.61–1.32)
	2, 4, 5, 7^f^				0.95	1.10 (0.92–1.31)
Tetralogy of Fallot	2, 5, 7, 9	655	0.23	1.03 (1.01–1.05)	< 0.001	0.80 (0.45–1.41)
	2, 5, 7, 10^e^	670	0.05	1.01 (0.99–1.04)	0.06	1.02 (0.75–1.39)
	2, 4, 5, 7^f^				0.13	1.13 (0.93–1.36)

a Studies included are different in each pollutant-anomaly meta-analysis depending on the data they published. References are as follows: 1, Ritz et al. (2002); 2, Gilboa et al. (2005); 4, Rankin et al. (2009); 5, Strickland et al. (2009); 6, Hansen et al. (2009); 7, Dolk et al. (2010); 9, Dadvand et al. (2011b); 10, Dadvand et al. (2011a).

b Number of cases included in the continuous exposure analysis.

c Conversion factors: CO, 1 ppb = 1.15 μg/m^3^; NO_2_, 1 ppb = 1.88 μg/m^3^; O_3_, 1 ppb = 1.96 μg/m^3^; SO_2_, 1 ppb = 2.62 μg/m^3^.

d When heterogeneity *p*-value is < 0.10, the OR from random effect model is shown; otherwise, the OR from the fixed-effects model is shown.

e Sensitivity analysis using Dadvand 2011a instead of Dadvand 2011b.

f Sensitivity analysis including Rankin 2009 instead of Dadvand 2011b.

**Table 3:** Summary of meta-analysis of studies on air pollutant exposures and orofacial clefts.

Pollutant and anomaly combination	Studies included^a^	Total number of cases^b^	Continuous exposure^c^		High versus low exposure	
			Heterogeneity *p*-value	Summary OR (95% CI)^d^	Heterogeneity *p*-value	Summary OR (95% CI)^d^
CO				per 1 ppm		

Cleft lip/palate^e^	1, 2, 3, 6, 8	1,498	0.03	0.89 (0.66–1.20)	0.002	0.99 (0.79–1.24)
Cleft palate	1, 2, 6, 8	697	0.009	0.68 (0.36–1.25)	0.06	0.78 (0.55–1.12)

NO_2_				per 10 ppb		

Cleft lip/palate	2, 6, 7, 8	1,423	0.44	0.99 (0.90–1.10)	0.25	1.06 (0.88–1.28)
Cleft palate	2, 6, 7, 8	809	0.29	0.98 (0.85–1.13)	0.23	0.79 (0.62–1.01)

O_3_				per 10 ppb		

Cleft lip/palate	1, 2, 3, 6, 8	1,950	0.20	1.10 (0.99–1.21)	0.22	1.06 (0.96–1.17)
Cleft palate	1, 2, 6, 8	702	0.79	0.99 (0.84–1.18)	0.42	1.00 (0.79–1.26)

PM_10_				per 10 μg/m^3^		

Cleft lip/palate	2, 3, 6, 7, 8	2,072	0.70	1.01 (0.96–1.06)	0.45	1.02 (0.94–1.11)
Cleft palate	2, 6, 7, 8	803	0.10	0.97 (0.82–1.16)	0.15	0.86 (0.70–1.07)

SO_2_				per 1 ppb		

Cleft lip/palate	2, 3, 6, 7, 8	1,976	0.06	0.99 (0.95–1.04)	0.01	1.06 (0.87–1.29)
	2, 3, 4, 6, 7, 8^f^				0.02	1.06 (0.89–1.27)
Cleft palate	2, 6, 7, 8	764	0.30	1.01 (0.99–1.03)	0.17	0.94 (0.81–1.10)

a References are as follows: 1, Ritz et al. (2002); 2, Gilboa et al. (2005); 3, Hwang and Jaakkola (2008); 4, Rankin et al. (2009); 6, Hansen et al. (2009); 7, Dolk et al. (2010); 8, Marshall et al. (2010).

b Number of cases included in the continuous exposure analysis.

c Conversion factors: CO, 1 ppb = 1.15 μg/m^3^; NO_2_, 1 ppb = 1.88 μg/m^3^; O_3_, 1 ppb = 1.96 μg/m^3^; SO_2_, 1 ppb = 2.62 μg/m^3^.

d When heterogeneity *p*-value is < 0.10 the OR from random effect model is shown; otherwise, the OR from the fixed-effect model is shown.

e Cleft lip with or without cleft palate.

f Sensitivity analysis including Rankin 2009 (no continuous exposure estimate available).
